# Is within-field spatial variation in wheat Fusarium crown rot associated with rhizosphere microbiome and soil nutrients

**DOI:** 10.3389/fpls.2026.1928197

**Published:** 2026-09-16

**Authors:** Fei Xu, Greg Deakin, Xiangming Xu

**Affiliations:** 1 Institute of Plant Protection, Henan Academy of Agricultural Sciences, Zhengzhou, China; 2 Key Laboratory of Integrated Pest Management on Crops in Southern Region of North China, Ministry of Agriculture and Rural Affairs of the People’s Republic of China, Zhengzhou, China; 3 Henan Key Laboratory of Agricultural Pest Monitoring and Control, Zhengzhou, China; 4 Xinxiang Observation and Research Station, Ministry of Agriculture and Rural Affairs, Xinixiang, China; 5 Niab East Malling, New Road, East Malling, Kent, United Kingdom

**Keywords:** Fusarium crown rot, *Fusarium pseudograminearum*, nitrate nitrogen, rhizosphere microbiome, soil physicochemical properties, spatial heterogeneity

## Abstract

Fusarium crown rot (FCR) of wheat, caused predominantly by *Fusarium pseudograminearum*, frequently exhibits marked spatial heterogeneity within individual fields despite uniform cultivars and agronomic management. However, the mechanisms underlying this within-field variation remain poorly understood. In this study, we investigated six commercial wheat fields in Henan Province, China, each containing distinct highly diseased (HD) and lightly diseased (LD) zones. Soil physicochemical properties and rhizosphere microbial communities (characterised via 16S rRNA and ITS amplicon sequencing) were compared between HD and LD zones. Despite identical management histories, HD and LD zones differed significantly in soil pH, nitrate nitrogen, available potassium, and magnesium. Furthermore, fungal communities in HD zones exhibited significantly lower α-diversity and distinct compositions, whereas bacterial communities showed only minor variations. *Fusarium* was significantly enriched in HD zones, while putatively beneficial taxa, including *Acremonium* and *Pseudoxanthomonas*, were more abundant in LD zones. These findings provide new insights into the micro-ecological factors associated with within-field disease heterogeneity and support the development of site-specific management strategies for FCR.

## 1 Introduction

Fusarium crown rot (FCR) of wheat, primarily caused by *Fusarium pseudograminearum*, is one of the most destructive soil-borne wheat diseases worldwide (Alahmad et al., 2018; Kazan and Gardiner, 2018; Pu et al., 2025). The pathogen initially infects leaf sheaths, resulting in a characteristic brown discoloration of the stem base (Cook, 1980). As the disease progresses, the fungus colonizes stem tissues, causing vascular blockage and extending upward into the higher internodes (Knight and Sutherland, 2016; Xu et al., 2024). Severe infections often lead to premature senescence, whitehead formation, shrivelled kernels, and substantial yield losses (Knight et al., 2017). On average, FCR causes yield losses of approximately 10%, though severe epidemics can result in > 50% losses (Smiley et al., 2005). In addition to yield loss, *F. pseudograminearum* can produce mycotoxins, such as deoxynivalenol (DON), in infected tissues and survive long-term in diseased crop residues (Fan et al., 2021; Xu et al., 2024). Once these infested residues are incorporated into the soil, pathogen inoculum accumulates over successive seasons, escalating the risk of severe disease outbreaks in subsequent wheat crops.

Many agronomic and environmental factors influence the occurrence and severity of FCR. Although host resistance is considered the most effective management strategy, highly resistant wheat germplasm remains limited; only a few cultivars, such as ‘Sunco’ and ‘2-49’ in Australia and ‘Zhengmai 7698’ in China, exhibit partial resistance (Liu and Ogbonnaya, 2015; Mitter et al., 2006; Wallwork et al., 2004; Xu et al., 2021). Nutrient management also significantly impacts FCR dynamics (Wang et al., 2023). Excessive nitrogen application has been shown to exacerbate crown rot severity and increase whitehead incidence (Buster et al., 2023; Cook, 1980; Paulitz, 2006), whereas phosphorus fertilization appears to have limited effect (Hagerty et al., 2021). Among micronutrients, zinc is associated with suppressed disease development, as zinc deficiency can enhance *F. pseudograminearum* colonization within wheat tissues (Grewal et al., 1996; Sparrow and Graham, 1988). Because *F. pseudograminearum* can persist in infected residues for up to two years, stubble management is critical (Wildermuth et al., 1997). Burning or deep tillage/incorporation of diseased residues significantly reduces inoculum levels (Burgess et al., 1996; Klein et al., 1988; Summerell and Burgess, 1988; Summerell et al., 1989). Similarly, precision sowing practices that minimize physical contact between stem bases and infested residues can decrease infection risk (Verrell et al., 2017). Finally, correct crop rotation can disrupt the disease cycle (Wildermuth et al., 1997). Continuous wheat cultivation or rotations with other cereal hosts accelerate pathogen accumulation and increase disease risk (Wallwork, 2005), whereas rotating with non-cereal crops can effectively suppresses inoculum buildup (Burgess et al., 1996; Felton et al., 1998; Fernandez and Zentner, 2005; Kirkegaard et al., 2004; Quazi et al., 2009). In addition, local variation in soil resource availability caused by competition with adjacent tree windbreaks has also been reported to influence the severity of wheat FCR (Smiley and MaChado, 2020).

Within-field FCR severity frequently exhibits pronounced spatial heterogeneity, even when the same cultivar is grown under the same management in the same field. Such localised variation suggests that small-scale environmental or biological factors significantly influence disease development, including soil moisture, nutrient availability, and soil (including rhizosphere) microbial communities. The rhizosphere microbiome, in particular, plays a critical role in mediating plant health and disease suppression (Rakhmatova et al., 2026; Zholdasbek et al., 2026). Accumulating evidence indicates that the diversity and composition of rhizosphere microbial communities are linked to host resistance against soil-borne pathogens (Wei et al., 2019, Wei et al., 2021). Generally, soils characterized by greater microbial diversity and complex community structures are more suppressive to pathogen invasion, whereas a disrupted rhizosphere microbiome can heighten disease susceptibility (Mallon et al., 2015). Consequently, characterising relationships between rhizosphere microbial dynamics and disease development is an important aspect of understanding correlation of within-field variability in FCR incidence and severity with micro-scale variation of relevant biological and environmental divers, ultimately facilitating the development of targeted, site-specific disease management strategies. To address this, the objective of this study was to characterize the distinct differences in rhizosphere microbial communities and soil physicochemical properties between severely and lightly diseased patches within a single, commercially managed wheat field.

## 2 Materials and methods

### 2.1 Experimental fields

Six commercial wheat fields exhibiting large within-field differences in FCR severity were selected in Henan Province, China. In each field, highly diseased zones and lightly diseased zones without FCR symptoms could be clearly distinguished, and the region of those fields is known for prevalent crown rot disease caused by *F. pseudograminearum* (Xu et al., 2026).

Two fields were in Yanjin County, Xinxiang City, Henan Province (35.1333°N, 114.2598°E, 59.5 m a.s.l.; 35.1315°N, 114.2624°E, 64.7 m a.s.l.) and were grown with the wheat cultivar ‘Xinmai 26’. Two fields were in Wen County, Jiaozuo City (34.9794°N, 112.9591°E, 105 m a.s.l.; 34.9801°N, 112.9608°E, 107 m a.s.l.) and were grown with the cultivar ‘Jindi 828’. The remaining two fields were in Neihuang County, Anyang City (36.1049°N, 114.9076°E, 43.9 m a.s.l.; 36.1039°N, 114.9073°E, 41.0 m a.s.l.) and were grown with the cultivar ‘Tianhe 6’. All these cultivars used in this study were susceptible to FCR. All the six fields followed a summer maize–winter wheat rotation. Following maize harvest, all maize residues were chopped and incorporated into the soil by rotary stubble cultivation before wheat sowing, ensuring complete residue return to the field. Wheat was sown in mid-October at a seeding rate of 187.5 kg ha^-1^ and harvested in early June next season. Basal fertilizers were applied at rates of 120 kg N ha^-1^, 90 kg P ha^-1^, and 90 kg K ha^-1^, followed by topdressing with 150–300 kg urea ha^-1^ at the jointing stage.

### 2.2 Disease assessment and sample collection

Each field was divided into a highly diseased zone (HD) and a lightly diseased zone (LD). Ten sampling points were established at each field, five in the HD zone and the other in the LD zones: at each sampling point, ten wheat tillers were collected and assessed for FCR on a 0–5 scale: 0 = no visible symptoms; 1 = discoloration restricted to the stem base; 2 = discoloration extending to the first node; 3 = discoloration extending to the second or third node; 4 = discoloration extending above the third node or the presence of whiteheads; and 5 = severely stunted or dead plants that failed to produce spikes. A composite disease index (DI) was calculated for each sampling point as the sum of the number of plants in each score class multiplied by the score) and then divided by 50 [namely =5 x 10].

Whitehead incidence was determined separately in HD and LD zones. Three 0.5 m^-2^ quadrats were randomly selected within each zone, and the numbers of whiteheads and total ears were recorded. For each HD and LD zone, wheat plants and inter-row soil samples were collected using an M-shaped sampling pattern from the five sampling points. Rhizosphere soil was used for microbial community analysis, whereas inter-row bulk soil was used for physicochemical analysis. Inter-row soil samples collected at the wheat grain-filling stage from the 0-15 cm soil layer at the five sampling points within each HD or LD zone were combined into a composite sample for soil physicochemical analysis, targeting the topsoil zone where wheat root activity, nutrient accumulation, and Fusarium crown rot primary infection predominantly occur (Barraclough and Leigh, 1984; Cook, 1980; Jobbágy and Jackson, 2001).

### 2.3 Rhizosphere soil sampling and microbial community sequencing

After transportation to the laboratory, soil loosely attached to roots was removed by gently shaking the roots. Soil tightly adhering to the root surface was then collected using sterilized brushes and transferred into sterile 1.5-mL microcentrifuge tubes. Sixty rhizosphere soil samples sent to Biomarker Technologies Corporation (Beijing, China) for amplicon sequencing: V3–V4 region of 16S rRNA gene for bacteria with the primers: 338F (5′-ACTCCTACGGGAGGCAGCA-3′), 806R (5′-GGACTACHVGGGTWTCTAAT-3′), and the fungal ITS1 region with ITS1F (5′-CTTGGTCATTTAGAGGAAGTAA-3′), and ITS2 (5′-GCTGCGTTCTTCATCGATGC-3′) primer pairs.

PCR amplification was conducted in a 25-μL reaction mixture containing 12.5 μL of 2× PCR Master Mix, 1 μL each of forward and reverse primers (10 μM), approximately 10 ng of template DNA, and nuclease-free water to the final volume. For 16S, PCR amplification was performed under the following conditions: initial denaturation at 98 °C for 2 min; 30 cycles of 98 °C for 30 s, 50 °C for 30 s, and 72 °C for 1 min; followed by a final extension at 72 °C for 5 min. For ITS, PCR consisted of an initial denaturation at 95 °C for 3 min; 30 cycles of 95 °C for 30 s, 55 °C for 30 s, and 72 °C for 45 s; followed by a final extension at 72 °C for 10 min.

A total of 5,438,406 paired-end reads were obtained from V3+V4 16S rRNA sequencing of 60 samples. After paired-end read merging and quality filtering, 4,589,552 clean tags were retained. Each sample generated at least 31,308 clean tags, with an average of 76,493 clean tags per sample (**Supplementary Table 1**). For ITS1 sequencing of the same 60 samples, 16,314,692 paired-end reads were generated. Merging and filtering yielded 13,835,424 clean tags. The minimum number of clean tags per sample was 116,484, and the average was 230,590 clean tags across samples (**Supplementary Table 2**).

### 2.4 Soil sampling and physicochemical analysis

For each treatment, five inter-row soil cores were thoroughly mixed to form a composite sample, and plant residues, roots, and stones were removed prior to sieving through a 2-mm mesh. A total of 12 composite soil samples (six fields × two disease severity levels) were analysed for the following soil properties: pH, organic matter content (OM), total carbon (C), available nitrogen (PMW), nitrate nitrogen (NO_2_^–^-N), ammonium nitrogen (NH_4_^+^-N), available phosphorus (P), available potassium (K), calcium (Ca), magnesium (Mg), iron (Fe), manganese (Mn), and zinc (Zn).

Soil at all six fields was classified as a Fluvo-aquic soil. For quantification of nitrate nitrogen and ammonium nitrogen, fresh soil samples were passed through a 2-mm sieve, sealed in plastic bags, stored at 4 °C, and analysed as soon as possible. Soil moisture content was determined. The remaining soil samples were air-dried for other analyses. Soil pH was measured potentiometrically in a soil-to-water suspension (1:2.5, w/v). Soil texture was determined using the hydrometer method. Soil organic matter content was quantified with the potassium dichromate oxidation method (Bao, 1981). Soil NO_2_^–^-N and NH_4_^+^-N were extracted with 2 mol L^–1^ KCl and quantified using a continuous flow analyser (AA3, SEAL Analytical, Germany). Permanganate oxidizable carbon (POXC) was quantified with the potassium permanganate oxidation method (Culman et al., 2012; Weil et al., 2003). Absorbance was measured at 550 nm with a UV–visible spectrophotometer (UV-2600, Shimadzu, Japan). Available phosphorus was extracted with 0.5 mol L^–1^ NaHCO_2_ (Olsen method) and quantified with the molybdenum blue colorimetric method. Available potassium was extracted with 1 mol L^–1^ NH_4_OAc and measured with a flame photometer (FP6410, Shanghai Precision & Scientific Instrument Co., Ltd., China). Exchangeable Ca and Mg were extracted with 1 mol L^–1^ NH_4_OAc (pH 7.0) and quantified with an inductively coupled plasma optical emission spectrometer (ICP–OES; iCAP 7400, Thermo Fisher Scientific, USA). DTPA-extractable Zn, Mn, and Fe were determined following DTPA extraction and quantified with an ICP–OES instrument (iCAP 7400, Thermo Fisher Scientific, USA). All analyses were conducted according to the procedures described by previous studies (Bao, 1981; Carter and Gregorich, 2022). Certified reference soil materials (GBW series, China) were included for quality assurance and quality control.

### 2.5 Bioinformatic and statistical analysis

All statistical analysis were carried out in R version 4.3.0. Permutational ANOVA [as implemented in the lmPerm package (Wheeler, 2016)] was applied to compare soil physiochemical traits between HD and LD samples with the field as a blocking factor. In Permutational ANOVA, all possible permutations ( n!) were calculated since there were only 12 samples. Soil physicochemical properties were analysed using zone-composited bulk soil samples (six pairs of HD-LD samples: one pair from each field), whereas rhizosphere microbiome data were obtained from 60 individual rhizosphere points (5 replicates per zone). The different sampling strategies preclude direct point-to-point correlation between bulk-soil physiochemical properties and rhizosphere microbiome, representing a methodological limitation of this study. However, it should be noted that in practice it was difficult to collate sufficient rhizosphere soils from wheat plants for physiochemical analysis.

Initial quality control was applied to raw sequence reads by the sequencing provider. Amplicon sequence variants (ASVs) were inferred using the DADA2 pipeline (v1.34) implemented in R (Callahan et al., 2016). Any ASVs with less than 11 total raw reads were excluded from further statistical analysis. Taxonomic assignment was carried out using the SINTAX algorithm implemented in USEARCH (Edgar, 2016). Fungal sequences were classified using a curated version of the Warcup V2 database, updated to align with NCBI taxonomy as of 2 September 2022. Bacterial taxonomy was assigned using the RDP training set version 18 for 16S rRNA gene sequences. A confidence threshold of 80% was applied for all taxonomic assignments.

The effect of disease classes (namely HD vs. LD) on alpha (α – within sample) and beta (β – between sample) diversity indices were assessed using ‘y ~ Field + Disease_Class’ where y is the dependent variable of interest. The R package vegan version 2.5-6 (Oksanen et al., 2019) was used to calculate α diversity indices: richness (ACE), and two diversity indices (Shannon and Simpson). Effects on a diversity indices were assessed by permutational ANOVA of the index ranks with the R package lmPerm v2.1.0 (Wheeler, 2016). Non-metric multidimensional scaling (NMDS) of the Bray-Curtis (β diversity) indices (of the DESeq2 normalised ASV count data) was carried out with vegan. Permutational multivariate analysis of variance (PERMANOVA) through the “adonis2” function in the vegan package was used to quantify treatment effects based on the marginal effect model. In addition, Principal Component Analysis (PCA) were applied to the normalised ASV counts data with the DESeq2 variance stabilising transformation method (Anders and Huber, 2010). As for diversity indices, ANOVA was then performed to quantify the effects of experimental factors to the observed variability in the PC scores. Finally, we used the umap R package (Konopka, 2018) to project and visualize rhizosphere microbiome data of all samples.

DESeq2 analyses were applied to compare the relative abundance of individual ASVs and aggregated taxonomic features between HD and LD samples within the framework of generalised linear modeling, assuming a negative binomial distribution for residuals (Love et al., 2014). ASVs were aggregated to higher taxonomic ranks prior to differential testing, ASVs with < 80% confidence in assigning a taxonomy identification were excluded. DeSeq2 analysis was applied to all taxonomy ranks for fungi (phylum, class, order, family, genus, species and ASV). For bacteria species-rank comparisons were not performed owing to unavailable species-level taxonomic assignments. The significance of differentially abundant features was based on a Wald test with the p-value adjusted by the Benjamini-Hochberg (BH) method (Benjamini and Hochberg, 1995).

Preliminary results indicated that *Podosphaera fuliginea*, an obligate foliar pathogen specific to cucurbit crops, dominated the fungal ASVs (approximately 12% of total fungal reads) in wheat rhizosphere soil. Ecologically, *P. fuliginea* is incapable of colonizing wheat roots or maintaining active indigenous populations in the rhizosphere. For this reason, several *Podosphaera fuliginea* ASVs were excluded from statistical analysis of α and β diversity indices., and differential abundance comparisons.

## 3 Results

### 3.1 Soil physicochemical properties differed between HD and LD zones

**Table 1** gives the summary of FCR disease index and whitehead incidence for HD and LD samples within each field, whereas average values of all measured soil properties across the six fields are given in **Table 2**. Permutational ANOVA revealed significant differences between HD-and LD-zone samples in pH (*P* < 0.01), NO_2_^–^-N (*P* < 0.001), available K (*P* < 0.01) and Zinc (*P *< 0.01), whereas the difference in Mg was close to achieving statistical significance (*P *= 0.057). Soils from HD zones had higher NO_2_^–^-N, and K relative to LD zones. By contrast, LD zones exhibited higher pH, Mg, and Zn (**Table 2**).

**Table 1:** Disease index (DI) of wheat Fusarium crown rot and whitehead incidence in highly diseased (HD) and lightly diseased (LD) zones within six wheat fields.

		Disease index (DI)		White heads	
Field	Site	HD	LD	HD	LD
18NHS1	Neihuang, Anyang	71.2±6.5	11.2±9.3	33%	10%
18NHS2	Neihuang, Anyang	53.6±6.9	35.2±5.1	38%	1.5%
18WXS1	Wenxian, Jiaozuo	40.0±9.7	8.8±4.3	50%	0%
18WXS2	Wenxian, Jiaozuo	37.6±5.9	3.2±1.6	45%	1%
18YJS1	Yanjing, Xinxiang	72.4±4.4	23.6±6.9	35%	1%
18YJS2	Yanjing, Xinxiang	64.0±9.4	8.4±4.9	14%	0.1%

**Table 2:** Average values of soil pH and nutrients for two composite samples in each field: one from five zones with a high level of FCR, and the other from five zones with low level of the disease.

Features	Highly diseased zone	Lightly diseased zone
pH	8.38±0.06^a^	8.43±0.03
OM (%)	1.70±0.19	1.72±0.17
C (%)	0.98±0.11	1.00±0.10
PMN (mg/kg)	61.04±7.35	58.80±9.18
NO_3_^-^-N (mg/kg)	15.16±3.56	9.30±2.13
NH_4_^+^-N (mg/kg)	3.23±0.50	4.30±1.52
P (mg/kg)	23.70±3.05	21.53±1.66
K (mg/kg)	293.93±40.37	251.32±30.36
Ca (mg/kg)	16836.41±1039.77	16953.96±1138.16
Mg (mg/kg)	994.28±195.21	1077.07±216.82
Fe (mg/kg)	2.97±0.30	3.01±0.45
Mn (mg/kg)	4.89±0.27	5.08±0.29
Zn (mg/kg)	1.28±0.17	1.44±0.19

^a^ Standard error is given in the bracket after ‘±’. In total, six fields were sampled.

### 3.2 General amplicon sequencing and taxonomy

All 60 samples yielded a sufficient number of high-quality sequence reads for downstream analyses. For fungal ITS sequencing, the number of raw reads per sample ranged from 112,630 to 380,786 (median = 203,672), resulting in 3,829 fungal amplicon sequence variants (ASVs). The number of raw reads per ASV ranged from 22 to 1,534,875 (median = 195). Successful taxonomic assignment at an 80% confidence threshold decreased from the phylum level (54% of ASVs; 88% of reads) to the species level (11% of ASVs; 28% of reads). Fungal communities were dominated by Ascomycota, with negligible differences between HD and LD samples (**Supplementary Figure S1A**). At the class level, Sordariomycetes was the dominant taxon (**Figure 1A**). Compared with the LD samples, HD samples contained a higher relative abundance of Leotiomycetes but lower abundances of Sordariomycetes and Dothideomycetes. The most abundant fungal ASV was identified as *Podosphaera fuliginea*, accounting for approximately 12% of total reads, followed by ASVs assigned to Sordariomycetes and *Fusarium*, which represented 10.0% and 7.5% of total reads, respectively.

**Figure 1:**
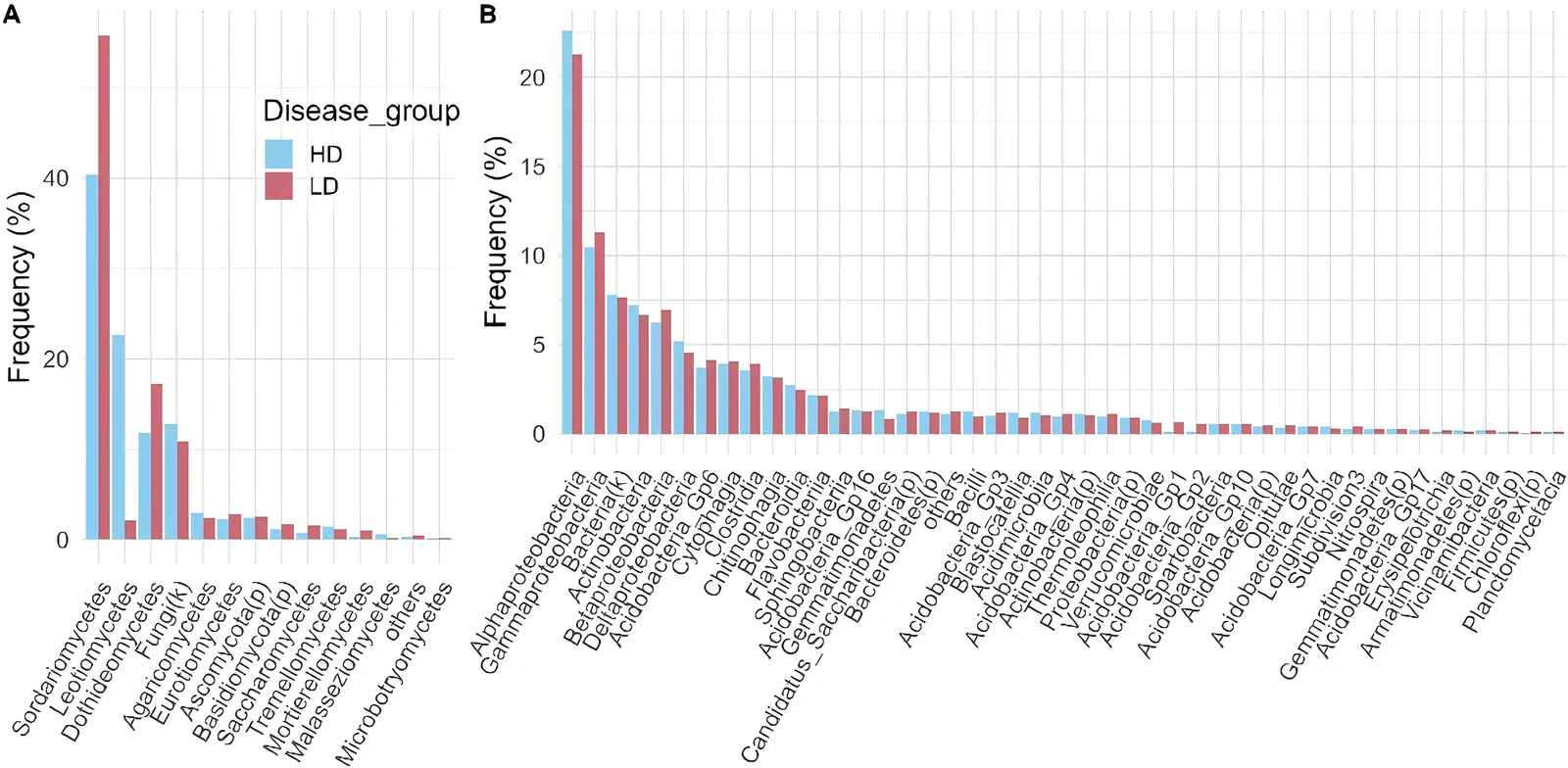
Proportion of raw reads in each class: fungi **(A)** and bacteria **(B)**. There were 60 wheat rhizosphere samples from six wheat fields; within each field, five samples were from five highly diseased (HD) zones (*Fusarium* crown rot), and the other five from five lightly diseased (LD) zones.

For bacterial 16S rRNA sequencing, the number of raw reads per sample ranged from 9,766 to 169,390 (median = 27,240), yielding a total of 27,572 bacterial ASVs. The number of raw reads per ASV ranged from 11 to 17,021 (median = 27). Successful taxonomic assignment varied from 87% of ASVs (92% of reads) at the phylum level to 41% of ASVs (53% of reads) at the genus level. Proteobacteria was the dominant bacterial phylum with negligible differences between HD and LD samples (**Supplementary Figure S1B**), while Alphaproteobacteria was the most abundant bacterial class (**Figure 1B**). The three most abundant bacterial ASVs were assigned to the genera *Enterocloster*, *Brucella*, and *Sphingomonas*, accounting for 1.1%, 0.6%, and 0.6% of total reads, respectively. Two additional *Sphingomonas* ASVs were also ranked among the six most abundant bacterial ASVs.

### 3.3 Within-sample (α diversity)

A summary of the statistical analysis for α-diversity indices is presented in **Table 3**. Permutational ANOVA revealed that HD and LD samples differed significantly across all three fungal α-diversity metrics: ACE ( P<0.01), Shannon ( P<0.001), and Simpson ( P<0.01) (**Table 3**). Overall, LD samples exhibited lower richness than HD samples (376 vs. 428) but higher Shannon (3.86 vs. 3.65) and Simpson (0.93 vs. 0.91) values (**Figure 2A**), indicating a more even distribution of fungal taxa in LD samples. This contrasting pattern indicates that HD zones harbour a greater number of rare low-abundance taxa (reflected by higher ACE richness), probably because the predominance by *Fusarium* in HD samples may have suppressed many other fungal taxa. Conversely, differences in bacterial α-diversity indices between HD and LD samples were limited (**Figure 2B**) and not statistically significant (**Table 3**).

**Table 3:** Percentage of variability (sum of squares) in each alpha (ACE, Shannon, Simpson) and beta (Bray-Curtis [BC]) diversity index and all principal components (PCs) accounted by each model term, based on marginal (Type III) sums of squares.

Terms	Fungi					Bacteria				
	ACE	Shannon	Simpson	BC	PCs^$^	ACE	Shannon	Simpson	BC	PCs
Disease Group	3.6^**^	8.7^***^	10.2^***^	5.7^***^	2.2	1.3	1.1	1.2	3.0^***^	2.2
Fields	61.3^***^	10.0^***^	10.5^**^	34.2^***^	25.4	19.5^**^	18.3^**^	18.3	30.0^***^	17.4
Residuals	35.0	82.7	79.2	60.1	72.7	79.2	80.6	80.5	67.0	80.4

^$^ Statistical significance is not given for between and within block differences and is also not available for the overall principal components (PCs): **, and *** represents *P* ≤ 0.01 and 0.001, respectively.

**Figure 2:**
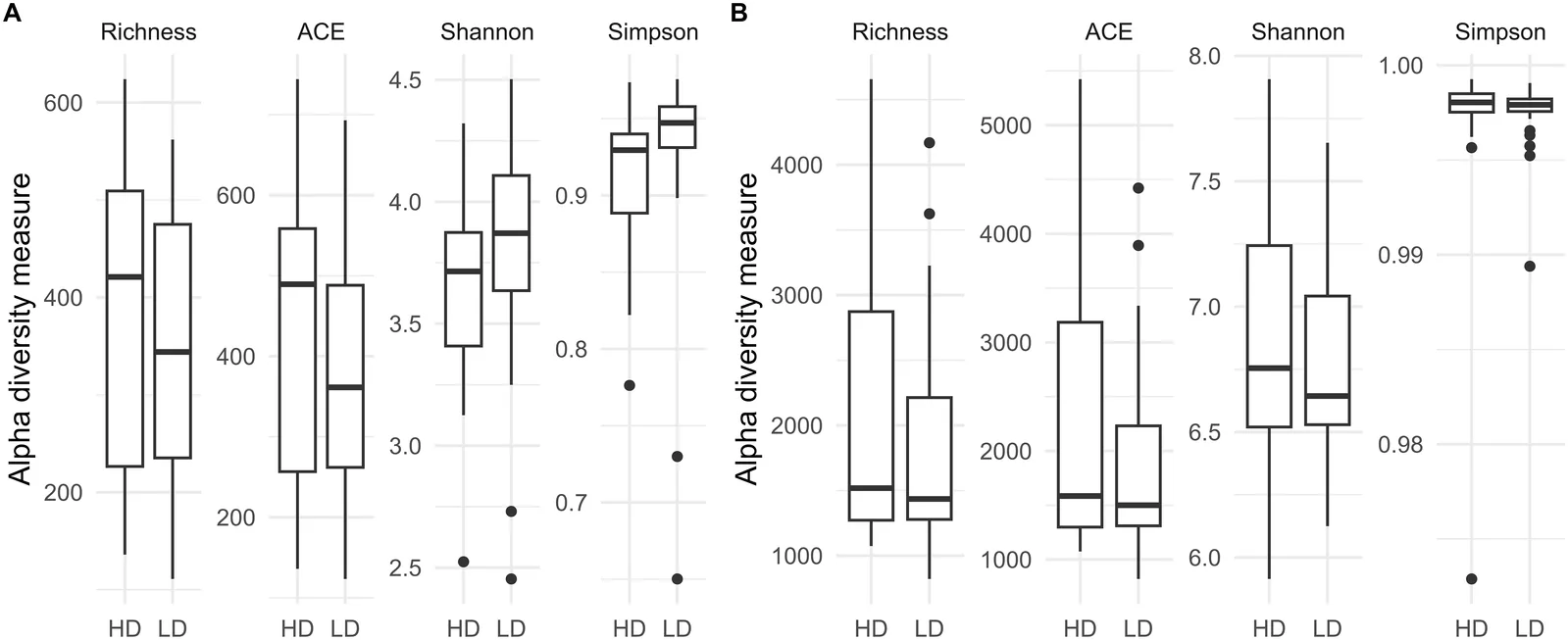
Alpha (a) diversity indices (ACE, Shannon, and Simpson): fungi **(A)** and bacteria **(B)**. There were 60 wheat rhizosphere samples from six wheat fields; within each field, five samples were from five highly diseased (HD) zones (*Fusarium* crown rot), and the other five from five lightly diseased (LD) zones.

### 3.4 Between sample (β-diversity)

Similar trends were observed for β-diversity indices as for α-diversity metrics. Specifically, variation among the six fields contributed substantially more to the overall variability in Bray-Curtis dissimilarity than did differences in disease severity groupings (**Table 3**).

Fungal Bray-Curtis indices differed significantly ( P<0.001) between HD and LD samples. Of the first four fungal principal components (PCs), only PC3 differed ( P<0.001) between HD and LD samples, whereas all four (PC1–PC4) varied significantly among the six fields ( P<0.001). Across all PCs, differences between HD and LD samples accounted for a mere 2.25% of the total variability, compared to 25.38% explained by variations among the six fields. Similarly, bacterial Bray-Curtis indices differed ( P<0.001) between HD and LD samples. None of the first four bacterial PCs varied significantly between HD and LD samples, although the differences for PC1, PC2, and PC4 approached statistical significance ( 0.05<P<0.10). Across all bacterial PCs, differences between HD and LD samples explained only 2.2% of the total variability, whereas differences among the six fields accounted for 17.4%. **Figure 3** illustrates these variations in the rhizosphere microbiome between the six fields and across HD and LD conditions.

**Figure 3:**
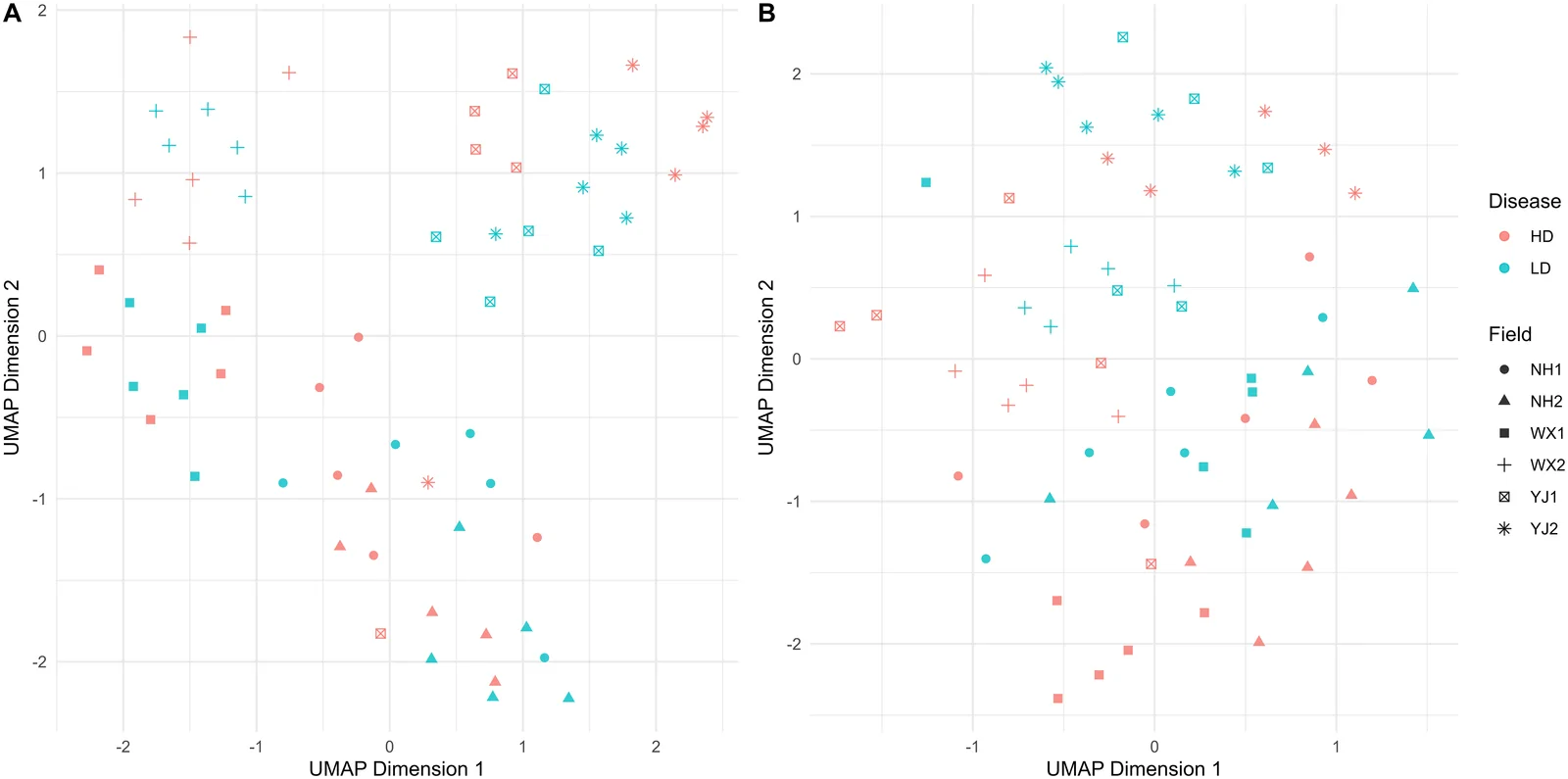
UMAP visualization of high-dimensional wheat rhizosphere fungal **(A)** and bacterial **(B)** microbiome data. The original microbiome data were projected onto a two-dimensional manifold using UMAP. There were 60 wheat rhizosphere samples from six wheat fields; within each field, five samples were from five highly diseased (HD) zones (Fusarium crown rot), and the other five from five lightly diseased (LD) zones.

### 3.5 Relative abundance of individual ASVs between HD and LD samples

Summary results of the differential abundance analysis across different taxonomic ranks are presented in **Table 4**. Overall, only a small proportion of fungal and bacterial taxa differed in relative abundance between HD and LD samples, particularly for bacteria. For instance, out of the 27,572 bacterial ASVs subjected to DESeq2 analysis, only 10 differed significantly in their relative abundance between HD and LD samples, compared to 156 out of 3,829 fungal ASVs. Only the most prominent differentially abundant ASVs are described below, whereas the complete dataset is presented in **Supplementary Tables 3**–**6**.

**Table 4:** Summary of DESeq2 analysis of differentially abundant fungal and bacterial taxa at different taxonomic ranks between highly diseased (HD) and lightly diseased (LD) rhizosphere soils from six wheat fields.

Treatment	Phylum	Class	Order	Family	Genus	Species*	ASV
Fungi	5 (0, 1)^$^	21 (0, 5)	53 (1, 5)	118 (2, 12)	198 (5, 11)	221 (7, 8)	3379 (74, 82)
Bacteria	35 (0, 0)	74 (1, 2)	107 (2, 3)	252 (4, 2)	639 (5, 5)		27572 (1, 9)

*For 16S, amplicon sequences were insufficient for assigning taxonomy to the species level.

^$^ x(y, z): x - total number of taxa groups compared; y and z - respective number of taxa groups with relative abundance increased or decreased at adjusted *P* value of 0.05.

One *Fusarium* ASV was highly enriched in HD samples ( LFC=1.87) with a high mean abundance of 24,573, compared to just 653 for the second most abundant ASV with differential abundance (**Supplementary Table 3**). The third most abundant ASV (mean abundance = 471) with differential abundance was also a *Fusarium* ASV enriched in HD samples (LFC = 5.64). Similarly, a *F. nygamai* ASV (mean abundance = 260) was strongly overrepresented in HD samples (LFC = 11.26). Additional ASVs enriched in HD samples included *Clonostachys epichloe*, *Gibellulopsis nigrescens*, *Myrmecridium schulzeri*, and *Candida parapsilosis*. In contrast, ASVs belonging to *Acremonium fusidioides*, *Saccharomycopsis fibuligera*, *Podospora tetraspora*, and *Thermoascus aurantiacus* were significantly more abundant in LD samples.

At the species level, the HD and LD samples differed in the relative abundance of 15 fungal species (**Supplementary Table 4**). Among these, three had high mean abundances (greater than 1,500), all of which were enriched in the LD samples: *Candida inconspicua*, *Acremonium fusidioides*, and *Dokmaia monthadangii*. At the genus rank, 15 fungal genera differed significantly in relative abundance between HD and LD samples. Noticably, *Fusarium* exhibited an exceptionally high mean abundance (31,952) and was significantly enriched in the HD samples. Conversely, higher abundances of *Acremonium*, *Dokmaia*, and *Pichia* were detected in the LD samples (**Supplementary Table 4**).

The relative abundances of only 10 bacterial ASVs differed significantly between HD and LD samples (**Supplementary Table 5**), all of which exhibited a low mean abundance (< 14). At the genus level, 10 bacterial genera varied in their relative abundance between HD and LD samples. Among these, only five had a mean abundance greater than 100 (**Supplementary Table 6**): *Acidibacter*, *Polaromonas*, Subdivision3_genera_incertae_sedis, *Pseudoxanthomonas*, and *Sorangium*. The first four genera were significantly more abundant in the LD samples, whereas *Sorangium* was less abundant in the LD group.

## 4 Discussion

In the present study, we investigated the relationship between within-field variation in FCR severity, soil physicochemical traits, and rhizosphere microbiome features. Our findings suggest that within-field variability in FCR may partially stem from specific soil physicochemical characteristics and, to a lesser extent, specific rhizosphere microbiome (particularly fungal) features. However, in interpreting these results, a specific limitation of the present study must be acknowledged: soil nutrient data were obtained from composite samples, which may not capture the fine-scale variation associated with individual sampling points (e.g., 10 stems). Future research utilizing high-resolution, point-specific sampling is warranted to better delineate these micro-environmental influences on disease severity.

Previous studies have demonstrated that soil nutrient availability is an important determinant of FCR development in wheat. Excessive nitrogen supply can exacerbate FCR development (Klein et al., 1991; Cook, 1980; Paulitz, 2006) and is associated with an increased incidence of whiteheads, possibly due to heightened late-season water stress (Cook, 1980; Papendick and Cook, 1974). In contrast, zinc (Zn) has been shown to suppress colonization by *F. pseudograminearum* and reduce FCR severity (Sparrow and Graham, 1988; Grewal et al., 1996). Our present findings align with these reports; specifically, soils from highly diseased zones contained higher levels of mineral nitrogen (PMN and ${\text{NO}}_{3}^{-}\text{-N}$) but lower concentrations of Zn than those from low-disease zones. Phosphorus levels appeared to have little effect on FCR development, which is consistent with the work of Hagerty et al. (2021). In addition, low-disease zones were characterized by higher pH and magnesium (Mg). Changes in soil pH can enhance the activity of *F. culmorum* cell wall-degrading enzymes, such as pectin lyase, while simultaneously suppressing host peroxidase activity, thereby facilitating pathogen colonization and reducing host defence capacity (Aleandri et al., 2008). Further research is needed to determine whether similar pH-related mechanisms apply to *F. pseudograminearum*. Furthermore, Mg is an essential macronutrient involved in chlorophyll biosynthesis, enzyme activation, and energy metabolism; adequate Mg nutrition may indirectly affect disease development by supporting plant vigour and influencing rhizosphere microbial processes (Huber and Jones, 2013). Mn may also be involved in plant defence-related metabolic pathways and contribute to plant–microbe interactions in the rhizosphere (Singh, 2015), though experimental evidence is required to support this hypothesis.

Previous studies on soil-borne diseases have frequently emphasized the importance of soil and rhizosphere bacterial communities in disease suppression (Garbeva et al., 2004; Mallon et al., 2015). However, the present study demonstrates that within-field FCR variability is more strongly associated with changes in fungal than bacterial communities. Fungal communities from highly diseased and low-disease zones differed significantly in both α- and β-diversity, whereas bacterial communities varied only in β-diversity. Moreover, a substantially greater number of fungal than bacterial ASVs differed in their relative abundance between these zones. Because FCR is a fungal soil-borne disease, interactions within the fungal rhizosphere community are inherently more likely to influence pathogen infection and subsequent colonization than interactions within the bacterial community. Overall, the reduced fungal community evenness, coupled with the enrichment of *Fusarium* in highly diseased zones, may have contributed to creating a rhizosphere environment conducive to pathogen dominance and FCR development.

Notably, several ASVs from *Podosphaera fuliginea*, an obligate foliar pathogen specific to cucurbit crops, dominated the fungal ASVs (approximately 12% of total fungal reads) in wheat rhizosphere soil. Ecologically, *P. fuliginea* is incapable of colonizing wheat roots or maintaining active indigenous populations in the rhizosphere via surviving saprophytically. Thus, its high abundance does not reflect functional rhizosphere colonisation, but is likely caused by exogenous dormant spores or contamination of *P. fuliginea* in aerial inoculum originating from adjacent cucurbit fields. The extensive cultivation of cucurbit crops during the wheat growing season could be associated with such elevated reads numbers, with aerial and rain-splash dispersal from infected neighboring tissues acting as a passive inoculum transmission pathway. ASVs from taxon were thus excluded from subsequent functional and disease-related analysis, as it represents an allochthonous environmental signal rather than a native component of wheat rhizosphere microbiome.

Rhizosphere microbial analysis further indicated that FCR disease severity was associated with numerous individual microbial taxa, particularly fungi. This aligns with the reduced fungal community evenness observed in the high disease (HD) samples. *Fusarium*-associated amplicon sequence variants (ASVs) were significantly correlated with the HD zones, including several highly abundant *Fusarium* ASVs and an ASV assigned to *F. nygamai*, which has been reported as a pathogen associated with FCR in wheat (Kharipzhanova et al., 2025). The genus *Fusarium* contains numerous wheat pathogens associated with crown rot, including *F. pseudograminearum* and *F. graminearum*, which are among the primary causal agents of FCR (Kazan and Gardiner, 2018; Xu et al., 2018). At the genus level, *Fusarium* abundance was substantially higher in HD zones. Although ASV-based analysis failed to resolve *Fusarium* ASVs at the species level, the enrichment of *Fusarium*-associated taxa in the rhizosphere of wheat plants from HD zones supports the status of FCR as a soil-borne disease caused by *Fusarium*. The high relative abundance of *Fusarium* could originate from the subsequent spread of the fungus following the initial infection of crown tissues, from direct root infection, and/or from high inoculum levels in the soil. Further research is needed to ascertain the exact source of this high *Fusarium* abundance in wheat rhizosphere.

ITS amplicon sequencing used in this study only provides genus-level taxonomic resolution for *Fusarium* and cannot precisely distinguish pathogenic species (e.g., *F. pseudograminearum*) from non-pathogenic endophytic or saprophytic *Fusarium* taxa. Therefore, the observed *Fusarium* relative abundance in this study represents genus-level compositional variation, rather than the absolute biomass of pathogenic strains. Future studies should incorporate species-specific qPCR assays targeting functional marker genes (e.g., TEF-1α, TRI101) to achieve accurate quantification of pathogenic *F. pseudograminearum* and further validate the observed disease-associated microbial patterns.

Conversely, a higher abundance of *Acremonium fusidioides* was associated with low disease development. *Acremonium* species have been reported as endophytic fungi that may contribute to plant health and stress tolerance (Wachowska and Głowacka, 2014). In contrast, the high abundance of *Clonostachys epichiloë* correlated with the HD zones is difficult to explain, given that *C. epichiloë* is a well-known biocontrol fungus capable of suppressing numerous soil-borne pathogens (Jensen et al., 2000). Finally, *Candida inconspicua* and *Dokmaia monthadangii* were significantly associated with the LD zones; however, their ecological roles in the wheat rhizosphere remain unclear.

Previous studies have frequently attributed soil suppressiveness against *Fusarium* diseases to beneficial bacterial genera, such as *Bacillus*, *Pseudomonas*, *Paenibacillus*, and *Streptomyces* (Todorović et al., 2023). In contrast, the present study found that within-field variation in FCR severity was associated predominantly with changes in the rhizosphere fungal community rather than the bacterial community. Only a small number of bacterial taxa differed significantly in relative abundance between the HD and LD zones. Among these, the genus *Pseudoxanthomonas* was associated with the LD zones. This genus can promote soil organic matter decomposition and nutrient cycling, thereby improving the rhizosphere environment; additionally, certain strains exhibit antagonistic activity against soil nematodes (Hu et al., 2019). *Acidibacter* and *Polaromonas* were also identified as common rhizosphere bacteria involved in nutrient cycling and environmental adaptation (Jiao et al., 2026; Okazaki et al., 2021). Conversely, *Sorangium*—a myxobacterial genus capable of producing diverse secondary metabolites (Kim and Yun, 2011)—was more abundant in the HD zones. However, this pattern may reflect a microbial response to increased pathogen pressure rather than a direct role in disease development.

Our variance-partitioning analysis showed that field locations explained a far larger proportion of rhizosphere bacterial and fungal community variation than disease group. This indicates that inherent edaphic and agronomic differences among commercial fields represent primary factors associated with rhizosphere microbiome variation, and these field-scale factors can outweigh within-field disease-related microbial structuring. Although overall community-level differentiation between HD and LD zones was modest at best, several key functional taxa, including the pathogen *Fusarium* and potentially beneficial genera (*Acremonium* and *Pseudoxanthomonas*), exhibited consistent abundance shifts across all six surveyed fields. Such repeated taxon-level responses across independent sites suggest these microbial signals are biologically robust, even in the presence of weak global community dissimilarity among HD and LD rhizosphere.

## 5 Conclusion

The present findings suggest that soil nutrient availability and specific rhizosphere microbial features jointly may contribute to fine-scale variability in FCR severity (**Supplementary Figure 2**). Specifically, higher mineral nitrogen availability, coupled with reduced levels of other potential elements such as Zn and Mg, may create favorable conditions for *F. pseudograminearum* and shift rhizosphere microbial communities toward favoring FCR development. In contrast, soils characterized by more balanced nutrient conditions and a more diverse, even microbial (particularly fungal) community may suppress pathogen establishment. Therefore, fine-scale management of the soil as a growing substrate is critically important to suppress disease development, improve crop yield, and enhance field uniformity, which, in turn, can further optimize crop management efficiency.

## Data Availability

The datasets presented in this study can be found in the Genome Sequence Archive (GSA). The names of the GSA and accession number(s) can be found below: CRA048135, https://ngdc.cncb.ac.cn/gsa/browse/CRA048135.

## Supplementary material

Supplementary Figure 1 Proportion of raw reads in each phylum: fungi **(A)** and bacteria **(B)**. There were 60 wheat rhizosphere samples from six wheat fields; within each field, five samples were from five zones with highly disease (HD) incidence of Fusarium crown rot, and the other five from lightly disease (LD) zones.

Supplementary Figure 2 A conceptual diagram showing the hypothesized relationships among soil nutrients, rhizosphere microbiome, *Fusarium* abundance, and disease severity. Solid arrows represent promoting effects, dashed arrows indicate inhibitory effects. Soil nutrients shape rhizosphere microbiome assembly, which further modulates the abundance of pathogenic *Fusarium*. The variation in *Fusarium* abundance ultimately drives plant disease development. Indirect pathways from soil nutrients to disease severity mediated by the rhizosphere microbiome are also illustrated.
