# Supplementary material for: Is within-field spatial variation in wheat Fusarium crown rot associated with rhizosphere microbiome and soil nutrients
Source: Front Plant Sci. 2026 Sep 16;17:1928197. doi: 10.3389/fpls.2026.1928197 (PMC13623864; doi:10.3389/fpls.2026.1928197)
Supplement: Supplementary file 1 [file Table3.docx]

| TABLE S3 Summary of those wheat rhizosphere fungal ASVs whose relative abundance was increased (with positive log2FoldChang [LFC] values) or decreased (with negative LFC values) in the samples from wheat plants with high incidence of Fusarium crown rot over the samples from low incidence if Fusarium crown rot in the same field. | | | | | |
| --- | --- | --- | --- | --- | --- |
| **ASV** | **Predicted taxonomy** | **Mean abundance** | | **LFC** | **Adjusted P-values** |
| OTU1 | *Fusarium*(g) | 24573.33 | 1.87 | | 0.0032 |
| OTU70 | *Clonostachys epichloe*(s) | 652.9 | 3.57 | | 0.012 |
| OTU145 | *Fusarium*(g) | 470.97 | 5.64 | | 0 |
| OTU61 | *Gibellulopsis nigrescens*(s) | 409.63 | 8.36 | | 0 |
| OTU166 | Ascomycota(p) | 383.82 | 4.69 | | 0.043 |
| OTU60 | *Myrmecridium schulzeri*(s) | 351.84 | 4.21 | | 5.00E-04 |
| OTU77 | Basidiomycota(p) | 308.28 | 5.49 | | 0 |
| OTU171 | *Fusarium nygamai*(s) | 260.23 | 11.28 | | 0 |
| OTU514 | *Myrmecridium schulzeri*(s) | 170.49 | 12.76 | | 0 |
| OTU373 | Basidiomycota(p) | 166.58 | 10.08 | | 0.0054 |
| OTU119 | Agaricales(o) | 154.01 | 7.43 | | 6.00E-04 |
| OTU302 | Fungi(k) | 145.85 | 10.7 | | 8.00E-04 |
| OTU222 | *Coprinopsis*(g) | 125.21 | 7.13 | | 0.0264 |
| OTU510 | *Candida parapsilosis*(s) | 123.22 | 9.12 | | 0.0124 |
| OTU278 | Dothideomycetes(c) | 105.43 | 8.3 | | 0.0017 |
| OTU160 | Hypocreales(o) | 92.03 | 9.89 | | 0 |
| OTU377 | Fungi(k) | 91.81 | 8.23 | | 0.0335 |
| OTU627 | Hypocreales(o) | 85.44 | 6.61 | | 0.0034 |
| OTU421 | Fungi(k) | 83.99 | 8.22 | | 0.0344 |
| OTU399 | Hannaella oryzae(s) | 83.68 | 5.34 | | 0.0091 |
| OTU249 | Fungi(k) | 82.14 | 9.7 | | 0.0068 |
| OTU312 | Ascomycota(p) | 61.86 | 7.82 | | 0.0384 |
| OTU432 | Agaricales(o) | 55.89 | 12.45 | | 3.00E-04 |
| OTU275 | *Hyalorbilia oviparasitica*(s) | 55.34 | 10.47 | | 0 |
| OTU283 | Agaricomycetes(c) | 54.08 | 7.77 | | 0.0496 |
| OTU529 | Fungi(k) | 48.78 | 10.59 | | 0.003 |
| OTU611 | Agaricales(o) | 46.53 | 19.14 | | 0 |
| OTU620 | Fungi(k) | 45.99 | 19.13 | | 0 |
| OTU792 | Ascomycota(p) | 41.11 | 25.41 | | 0 |
| OTU335 | Sordariomycetes(c) | 40.08 | 12.37 | | 0 |
| OTU437 | Agaricales(o) | 38.16 | 19.81 | | 0 |
| OTU736 | Sordariomycetes(c) | 35.88 | 7.48 | | 0.0344 |
| OTU438 | Agaricales(o) | 35.51 | 7.78 | | 0.0344 |
| OTU425 | Fungi(k) | 26.45 | 4.02 | | 0.0427 |
| OTU945 | Ascomycota(p) | 25.67 | 18.68 | | 0 |
| OTU589 | Sordariales(o) | 24.33 | 6.83 | | 0.0233 |
| OTU1208 | Ascomycota(p) | 21.15 | 18.54 | | 0 |
| OTU902 | Fungi(k) | 19.42 | 9.61 | | 1.00E-04 |
| OTU687 | Psathyrellaceae(f) | 19.33 | 19.37 | | 0 |
| OTU869 | Fungi(k) | 17.12 | 7.9 | | 0.0447 |
| OTU1116 | Basidiomycota(p) | 16.64 | 7.37 | | 0.009 |
| OTU896 | Sordariales(o) | 15.94 | 24.46 | | 0 |
| OTU1425 | Fungi(k) | 15.93 | 18.56 | | 0 |
| OTU1601 | Podosporaceae(f) | 15.5 | 9.76 | | 0 |
| OTU1366 | Ascomycota(p) | 12.36 | 24.1 | | 0 |
| OTU781 | Fungi(k) | 12.15 | 18.81 | | 0 |
| OTU808 | *Podospora tetraspora*(s) | 10.71 | 10.12 | | 0.0039 |
| OTU1411 | Ascomycota(p) | 10.13 | 25.07 | | 0 |
| OTU778 | *Podospora tetraspora*(s) | 10.06 | 18.87 | | 0 |
| OTU834 | Ascomycota(p) | 9.59 | 18.51 | | 0 |
| OTU973 | Basidiomycota(p) | 9.51 | 23.97 | | 0 |
| OTU1418 | Fungi(k) | 9.46 | 23.56 | | 0 |
| OTU934 | Hypocreales(o) | 9.36 | 4.84 | | 0.0148 |
| OTU1048 | Basidiomycota(p) | 9.28 | 9.5 | | 4.00E-04 |
| OTU1476 | Basidiomycota(p) | 9.22 | 23.49 | | 0 |
| OTU1530 | Fungi(k) | 8.8 | 18.07 | | 0 |
| OTU889 | Psathyrellaceae(f) | 8.62 | 7.61 | | 0.0496 |
| OTU931 | Hypocreales(o) | 8.08 | 10.9 | | 0.0021 |
| OTU988 | Sordariomycetes(c) | 8 | 10.96 | | 5.00E-04 |
| OTU1071 | *Podospora bulbillosa*(s) | 7.77 | 9.82 | | 8.00E-04 |
| OTU1155 | *Panaeolus*(g) | 7.74 | 18.46 | | 0 |
| OTU1078 | Fungi(k) | 7.24 | 27.17 | | 0 |
| OTU1753 | Fungi(k) | 6.85 | 23.19 | | 0 |
| OTU1032 | *Fusarium haematococcum*(s) | 6.74 | 8.89 | | 0.0012 |
| OTU1442 | Basidiomycota(p) | 6.56 | 18.59 | | 0 |
| OTU2036 | [*Candida]* *intermedia*(s) | 6.36 | 17.85 | | 0 |
| OTU1246 | Agaricomycetes(c) | 5.15 | 18.25 | | 0 |
| OTU1435 | *Metarhizium flavoviride*(s) | 5.13 | 18.17 | | 0 |
| OTU1352 | Fungi(k) | 5.12 | 23.94 | | 0 |
| OTU1451 | Fungi(k) | 5.1 | 6.84 | | 0.0013 |
| OTU1754 | Fungi(k) | 4.76 | 9.19 | | 0.0022 |
| OTU1188 | Pleosporales(o) | 4.42 | 18.29 | | 0 |
| OTU1300 | Ascomycota(p) | 3.99 | 18.18 | | 0 |
| OTU1629 | *Mortierella alpina*(s) | 3.46 | 17.97 | | 0 |
| OTU1996 | Fungi(k) | 3.2 | 7.97 | | 0.0244 |
| OTU13 | *Acremonium fusidioides*(s) | 1838.68 | -2.39 | | 0.0091 |
| OTU15 | Ascomycota(p) | 1710.65 | -1.68 | | 0.0368 |
| OTU18 | Sordariomycetes(c) | 1517.93 | -2.53 | | 0.0348 |
| OTU68 | *Saccharomycopsis fibuligera*(s) | 778.39 | -3.02 | | 0.0117 |
| OTU110 | *Podospora tetraspora*(s) | 642.8 | -11.01 | | 0.0018 |
| OTU132 | Lasiodiplodia(g) | 577.09 | -10.76 | | 0 |
| OTU173 | *Thermoascus aurantiacus*(s) | 500.91 | -6.1 | | 0.0057 |
| OTU137 | Sordariomycetes(c) | 452.86 | -5.9 | | 8.00E-04 |
| OTU185 | Sordariales(o) | 409.22 | -7.81 | | 0.0276 |
| OTU144 | [*Candida*] *inconspicua*(s) | 369.51 | -4.46 | | 0.0024 |
| OTU203 | Fungi(k) | 309.67 | -5.62 | | 0.0181 |
| OTU308 | Sordariomycetes(c) | 298.12 | -13.65 | | 0 |
| OTU106 | Leotiomycetes(c) | 188.78 | -2.72 | | 0.009 |
| OTU393 | Fungi(k) | 164.35 | -10.12 | | 0.0052 |
| OTU118 | Sordariomycetes(c) | 134.74 | -5.36 | | 0.0115 |
| OTU506 | Fungi(k) | 130.23 | -25.05 | | 0 |
| OTU408 | Diaporthe(g) | 128.55 | -6.77 | | 0.043 |
| OTU371 | Basidiomycota(p) | 125.76 | -6.17 | | 0.0104 |
| OTU578 | Fungi(k) | 124.71 | -20.09 | | 0 |
| OTU287 | *Conocybe apala*(s) | 112.28 | -27.7 | | 0 |
| OTU164 | *Periconia macrospinosa*(s) | 97.1 | -8.62 | | 0 |
| OTU548 | Sordariales(o) | 93.5 | -30.27 | | 0 |
| OTU832 | *Leotia atrovirens*(s) | 85.77 | -19.81 | | 0 |
| OTU293 | Mortierellaceae(f) | 77.19 | -8.38 | | 0.0299 |
| OTU241 | Sordariomycetes(c) | 72.96 | -12.15 | | 0 |
| OTU513 | Agaricales(o) | 65.84 | -12.34 | | 3.00E-04 |
| OTU1047 | Orbiliaceae(f) | 62.42 | -11.08 | | 0.0017 |
| OTU235 | *Podila minutissima*(s) | 59.87 | -5.66 | | 0.0017 |
| OTU264 | Agaricomycetes(c) | 59.84 | -9.57 | | 0.0093 |
| OTU257 | Hypocreales(o) | 59.6 | -11.26 | | 0.0013 |
| OTU196 | Xylariales(o) | 59.24 | -4.98 | | 0.0299 |
| OTU813 | Sordariomycetes(c) | 57.27 | -7.39 | | 0.0177 |
| OTU460 | Fungi(k) | 55.72 | -8.33 | | 0.0306 |
| OTU567 | *Cyphellophora*(g) | 55.29 | -5.23 | | 0.0132 |
| OTU685 | *Conocybe apala*(s) | 54.03 | -10.59 | | 0.0028 |
| OTU830 | Ascomycota(p) | 49.5 | -7.85 | | 0.0464 |
| OTU638 | Fungi(k) | 40.81 | -13.02 | | 1.00E-04 |
| OTU305 | Agaricales(o) | 38.31 | -20.15 | | 0 |
| OTU651 | Fungi(k) | 34.49 | -19.35 | | 0 |
| OTU508 | Glomeraceae(f) | 34.24 | -12.43 | | 3.00E-04 |
| OTU633 | Fungi(k) | 32.97 | -7.94 | | 2.00E-04 |
| OTU347 | Stachybotryaceae(f) | 30.33 | -7.44 | | 0.0057 |
| OTU517 | Fungi(k) | 27.51 | -4.59 | | 0.0231 |
| OTU850 | Fungi(k) | 26.32 | -11.16 | | 0.0016 |
| OTU694 | Fungi(k) | 23.85 | -4.28 | | 0.0464 |
| OTU1730 | *Aspergillus penicillioides*(s) | 23.61 | -25.68 | | 0 |
| OTU572 | Chaetomiaceae(f) | 23.33 | -5.18 | | 0.0206 |
| OTU769 | *Hirsutella rhossiliensis*(s) | 23.2 | -10.03 | | 7.00E-04 |
| OTU800 | Fungi(k) | 22.52 | -8.88 | | 1.00E-04 |
| OTU840 | Fungi(k) | 22 | -18.79 | | 0 |
| OTU806 | *Zopfiella marina*(s) | 21.16 | -11.72 | | 8.00E-04 |
| OTU1083 | Fungi(k) | 18.02 | -10.33 | | 0.004 |
| OTU739 | Fungi(k) | 17.64 | -11.52 | | 8.00E-04 |
| OTU574 | *Mortierella*(g) | 17.29 | -7.8 | | 0.0041 |
| OTU819 | Fungi(k) | 15.9 | -8.74 | | 0.0221 |
| OTU2116 | Fungi(k) | 15.85 | -25.68 | | 0 |
| OTU943 | *Podospora tetraspora*(s) | 15.73 | -10.33 | | 0.004 |
| OTU909 | Ascomycota(p) | 14.25 | -8.25 | | 0.0344 |
| OTU935 | Fungi(k) | 13.29 | -5.93 | | 0.0182 |
| OTU767 | Fungi(k) | 13.25 | -11.62 | | 9.00E-04 |
| OTU717 | Sordariomycetes(c) | 12.71 | -10.27 | | 0.0043 |
| OTU1646 | Mucoromycota(p) | 12 | -24.61 | | 0 |
| OTU817 | Fungi(k) | 11.99 | -12.3 | | 4.00E-04 |
| OTU846 | Fungi(k) | 11.12 | -7.81 | | 0.0233 |
| OTU1383 | *Podospora tetraspora*(s) | 10.34 | -27.11 | | 0 |
| OTU814 | Sordariomycetes(c) | 9.91 | -8.01 | | 0.0358 |
| OTU1143 | Didymellaceae(f) | 9.36 | -8.14 | | 0.0365 |
| OTU859 | Fungi(k) | 8.86 | -9.77 | | 0.0074 |
| OTU947 | Fungi(k) | 8.66 | -8.55 | | 0.0037 |
| OTU1025 | Fungi(k) | 7.55 | -18.91 | | 0 |
| OTU1903 | Fungi(k) | 6.57 | -8.6 | | 0.0249 |
| OTU1596 | Hypocreales(o) | 6.54 | -7.16 | | 0.0172 |
| OTU1860 | S*arocladium*(g) | 6.37 | -27.75 | | 0 |
| OTU1693 | Microascales(o) | 4.8 | -18.66 | | 0 |
| OTU1375 | Fungi(k) | 4.57 | -6.39 | | 0.0066 |
| OTU1228 | Fungi(k) | 4.37 | -9.97 | | 0.006 |
| OTU1765 | Fungi(k) | 3.94 | -8.16 | | 0.0358 |
| OTU1785 | Fungi(k) | 3.3 | -18.23 | | 0 |
| OTU1855 | Fungi(k) | 2.79 | -18.28 | | 0 |
| OTU1824 | Fungi(k) | 2.53 | -7.94 | | 0.0441 |
| OTU1752 | *Solicoccozyma aeria*(s) | 2.46 | -18.18 | | 0 |
