# Supplementary material for: Is within-field spatial variation in wheat Fusarium crown rot associated with rhizosphere microbiome and soil nutrients
Source: Front Plant Sci. 2026 Sep 16;17:1928197. doi: 10.3389/fpls.2026.1928197 (PMC13623864; doi:10.3389/fpls.2026.1928197)
Supplement: Supplementary file 2 [file Table4.docx]

| TABLE S4 Summary of those wheat rhizosphere fungal species or genera whose relative abundance was increased (with positive log2FoldChang [LFC] values) or decreased (with negative LFC values) in the samples from wheat plants with high incidence of Fusarium crown rot over the samples from low incidence if Fusarium crown rot in the same field. | | | | | | |
| --- | --- | --- | --- | --- | --- | --- |
| **Predicted taxonomy** | **Mean abundance** | **LFC** | | **Adjusted P-values** | |  |
| *Species* |  |  |  | |  |  |
| *Clonostachys epichloe* | 652.90 | 3.57 | 0.003693 | |  |  |
| *Clonostachys rosea* | 227.57 | 2.82 | 0.0268 | |  |  |
| *Candida parapsilosis* | 123.22 | 9.16 | 0.017084 | |  |  |
| *Hyalorbilia oviparasitica* | 55.34 | 9.86 | 1.03E-13 | |  |  |
| *Edenia gomezpompae* | 55.07 | 4.081 | 0.002149 | |  |  |
| *Fusarium haematococcum* | 6.74 | 8.52 | 0.00059 | |  |  |
| *Metarhizium flavoviride* | 5.13 | 18.17 | 1.93E-08 | |  |  |
| *Dokmaia monthadangii* | 2323.56 | -1.13 | 0.019937 | |  |  |
| *Acremonium fusidioides* | 1838.68 | -2.39 | 0.002149 | |  |  |
| [*Candida*] *inconspicua* | 1735.49 | -2.72 | 0.002149 | |  |  |
| *Saccharomycopsis fibuligera* | 778.39 | -2.99 | 0.002481 | |  |  |
| *Periconia macrospinosa* | 167.66 | -2.55 | 0.049563 | |  |  |
| *Neosetophoma cerealis* | 114.43 | -2.32 | 0.043697 | |  |  |
| *Conocybe apala* | 112.28 | -27.70 | 3.70E-19 | |  |  |
| *Hirsutella rhossiliensis* | 23.20 | -9.85 | 0.00059 | |  |  |
| *Genus* |  |  |  | |  |  |
| *Fusarium* | 31952.83 | 1.31 | 0.000187 | |  |  |
| *Clonostachys* | 880.46 | 2.86 | 0.002705 | |  |  |
| *Hyalorbilia* | 55.34 | 9.83 | 1.80E-13 | |  |  |
| *Edenia* | 55.07 | 4.08 | 0.002705 | |  |  |
| *Knufia* | 9.02 | 4.73 | 0.002705 | |  |  |
| *Dokmaia* | 2323.56 | -1.13 | 0.019715 | |  |  |
| *Acremonium* | 2270.40 | -1.52 | 0.016305 | |  |  |
| *Pichia* | 1790.87 | -2.71 | 0.001883 | |  |  |
| *Linnemannia* | 938.36 | -2.22 | 0.021276 | |  |  |
| *Saccharomycopsis* | 778.39 | -2.99 | 0.002705 | |  |  |
| *Kazachstania* | 221.91 | -7.68 | 0.001511 | |  |  |
| *Bullera* | 176.65 | -1.15 | 0.002705 | |  |  |
| *Leotia* | 85.77 | -19.81 | 9.53E-10 | |  |  |
| *Podila* | 67.80 | -3.99 | 0.009995 | |  |  |
| *Lichtheimia* | 52.66 | -10.73 | 0.003062 | |  |  |
