# Supplementary material for: Is within-field spatial variation in wheat Fusarium crown rot associated with rhizosphere microbiome and soil nutrients
Source: Front Plant Sci. 2026 Sep 16;17:1928197. doi: 10.3389/fpls.2026.1928197 (PMC13623864; doi:10.3389/fpls.2026.1928197)
Supplement: Supplementary file 4 [file Table6.docx]

| TABLE S6 Summary of those wheat rhizosphere bacterial genera whose relative abundance was increased (with positive log2FoldChang [LFC] values) or decreased (with negative LFC values) in the samples from wheat plants with high incidence of Fusarium crown rot over the samples from low incidence if Fusarium crown rot in the same field. | | | | | |
| --- | --- | --- | --- | --- | --- |
| **Predicted taxonomy** | **Mean abundance** | | **LFC** | **Adjusted P-values** |  |
| *Sorangium* | 100.41 | 1.43 | | 0.001006 |  |
| *Taibaiella* | 60.78 | 1.31 | | 0.010279 |  |
| *Agaricicola* | 26.26 | 2.28 | | 0.011173 |  |
| *Herpetosiphon* | 13.75 | 1.50 | | 0.010279 |  |
| *Nitrosospira* | 11.34 | 2.76 | | 0.023202 |  |
| *Pseudoxanthomonas* | 309.17 | -0.52 | | 0.049956 |  |
| *Acidibacter* | 120.95 | -1.29 | | 0.000869 |  |
| *Polaromonas* | 113.94 | -0.98 | | 0.008252 |  |
| *Subdivision3_genera_incertae_sedis* | 113.04 | -0.75 | | 0.010279 |  |
| *Lacibacter* | 12.62 | -3.34 | | 0.000549 |  |
